# Prediction of organic compound aqueous solubility using machine learning: a comparison study of descriptor-based and fingerprints-based models

**DOI:** 10.1186/s13321-023-00752-6

**Published:** 2023-10-18

**Authors:** Arash Tayyebi, Ali S Alshami, Zeinab Rabiei, Xue Yu, Nadhem Ismail, Musabbir Jahan Talukder, Jason Power

**Affiliations:** 1 University of North Dakota, Chemical Engineering, Grand Forks, ND 58201 USA; 2 Chemistry Department, University of North Dakota, Grand Forks, ND 58202 USA; 3 Energy & Environmental Research Center, University of North Dakota, Grand Forks, ND 58202 USA; 4 University of North Dakota, Biomedical Sciences, Grand Forks, ND 58202 USA

**Keywords:** Aqueous solubility, Fingerprint, Machine learning, Random forest, SHAP

## Abstract

A reliable and practical determination of a chemical species’ solubility in water continues to be examined using empirical observations and exhaustive experimental studies alone. Predictions of chemical solubility in water using data-driven algorithms can allow us to create a rationally designed, efficient, and cost-effective tool for next-generation materials and chemical formulations. We present results from two machine learning (ML) modeling studies to adequately predict various species’ solubility using data for over 8400 compounds. Molecular-descriptors, the most used method in previous studies, and Morgan fingerprint, a circular-based hash of the molecules' structures, were applied to produce water solubility estimates. We trained all models on 80% of the total datasets using the Random Forest (RFs) technique as the regressor and tested the prediction performance using the remaining 20%, resulting in coefficient of determination (R^2^) test values of 0.88 and 0.81 and root-mean-square deviation (RMSE) test values 0.64 and 0.80 for the descriptors and circular fingerprint methods, respectively. We interpreted the produced ML models and reported the most effective features for aqueous solubility measures using the Shapley Additive exPlanations (SHAP) and thermodynamic analysis. Low error, ability to investigate the molecular-level interactions, and compatibility with thermodynamic quantities made the fingerprint method a distinct model compared to other available computational tools. However, it is worth emphasizing that physicochemical descriptor model outperformed the fingerprint model in achieving better predictive accuracy for the given test set.

**Supplementary Information:**

The online version contains supplementary material available at 10.1186/s13321-023-00752-6.

## Introduction

Chemical compounds solubility in various solvents is one of the most important properties for understanding the physicochemical behavior of various materials and chemical formulations, as well as the design and synthesis of the next-generation materials. Aqueous solubility predictions have been the subject of numerous research and application studies, ranging from environmental predictions, biochemistry, chemical process design, and agrochemical uses to drug development [1, 2]. Solubility prediction remains a critical challenge due to the lack of reliable and reproducible measurements alongside the time and cost of experimental approaches. A machine learning (ML) algorithm that accurately describes behavioral component compositions can be used to fulfill this requirement. ML techniques will allow us to test a significant majority of materials without a physical sample and more efficiently determine the materials’ physical properties, such as solubility. The primary challenge when using ML algorithms for aqueous solubility predictions is that the solubility experimental data are most often unreliable, and the resultant models would be no better than the raw data. Nonetheless, using a large database can greatly improve a model’s accuracy and reliability.

Several computational models capable of predicting a molecule’s aqueous solubility have been reported in the literature. Descriptor-based [3–6], and group contribution [7–9] are two distinct data preparation approaches that have been used recently in various modeling methods to determine solubility measures. Parameters in descriptor-based models rely on physical properties such as molecular topological indices. Group contribution methods demonstrate a correlation between water solubility and several functional groups by decomposing the molecular units into subunits and adding the estimated solubility of each of these subunits together. A comparison between notable developed models and their performance is presented in Table 1.

**Table 1:** Comparison between different current models that predict water solubility

Developer	Data Preparation Method	Total Size	ML Method	R^2^ Test Value^7^	MAE^8^	RMSE^9^	SEP^10^	Refs
Huuskonen	Descriptor-Based	1297	MLR^1^	0.88	–	0.71	–	[10]
			ANN^3^	0.92	–	0.60	–	
Yan	Descriptor-Based	1293	MLR	0.82	0.68	0.79	–	[11]
			ANN	0.96	0.49	0.59	–	
Delaney	Descriptor-Based	2874	MLR	0.71	0.68	0.87	–	[12]
Hou	Group Contribution	1294	MLR	0.9	0.52	0.63	–	[2]
Ali	Descriptor-Based	1290	MLR	0.73	0.72	0.94	–	[13]
Sorkun	Descriptor-Based	1290	Ensemble of ANN, RF^2^, and XGB^4^	0.93	0.397	0.53	–	[14]
Le	Descriptor-Based	4376	MLR	0.89	–	–	0.75	[15]
			MLREM^5^	0.88	–	–	0.76	
			BRANNLP^6^	0.90	–	–	0.66	

Total size in this table stands for the number of datasets used to train each of the algorithms

^1^MLR: Multilinear Regression; ^2^RF: Random Forest; ^3^ANN: Artificial Neural Network; ^4^XGB: Gradient Boosted Trees; ^5^MLREM: multiple linear regression with expectation maximization; ^6^BRANNLP: Bayesian regularized artificial neural network with a Laplacian prior; ^7^R^2^: squared coefficient of determination; ^8^MAE: mean absolute error; ^9^RMSE: root-mean-square deviation; ^10^SEP: standard error of prediction

Previous studies have revealed that aqueous solubility prediction is accessible; however, researchers new to the field may face difficulties in comprehending these algorithms due to their complex physicochemical characteristics. Moreover, most concerns with current studies are related to the validity of the correlations (since they are very susceptible to the variations of the conditions used during calibration) where they are defined ahead of time. Also, the effect of chemical representations and their role in a ML method’s performance have not been thoroughly investigated.

In this study, we compared the descriptor-based and fingerprint methods for investigating the effects of data preparation ahead and behind the time on the ML’s accuracy. The fingerprint model used in this study is similar to the group contribution methods addressed above, with the advantage that it does not obtain the chemical building blocks in advance. Additionally, the fingerprint model is derived from physicochemical insights [16], which allows for easier interpretation of the model, and is useful in the context of developing efficient Quantitative Structure–Property Relationships (QSPRs) for the solubility [17]. The significance of this study lies in the practical utility of the developed fingerprint model, which can aid experts in investigating the impact of different functional groups on solubility predictions, which can have important implications for drug discovery and other related applications.

## Materials & method

### Data acquisition

The data needed for model training are vital if the model is to interpret many aspects, including feature selection effectiveness, applicability domain, and ability to handle the various contributions that can describe the equilibrium between the solute’s dissolved and bulk states. A significant amount of data will lead to reliable data-driven models.

**Fig. 1:**
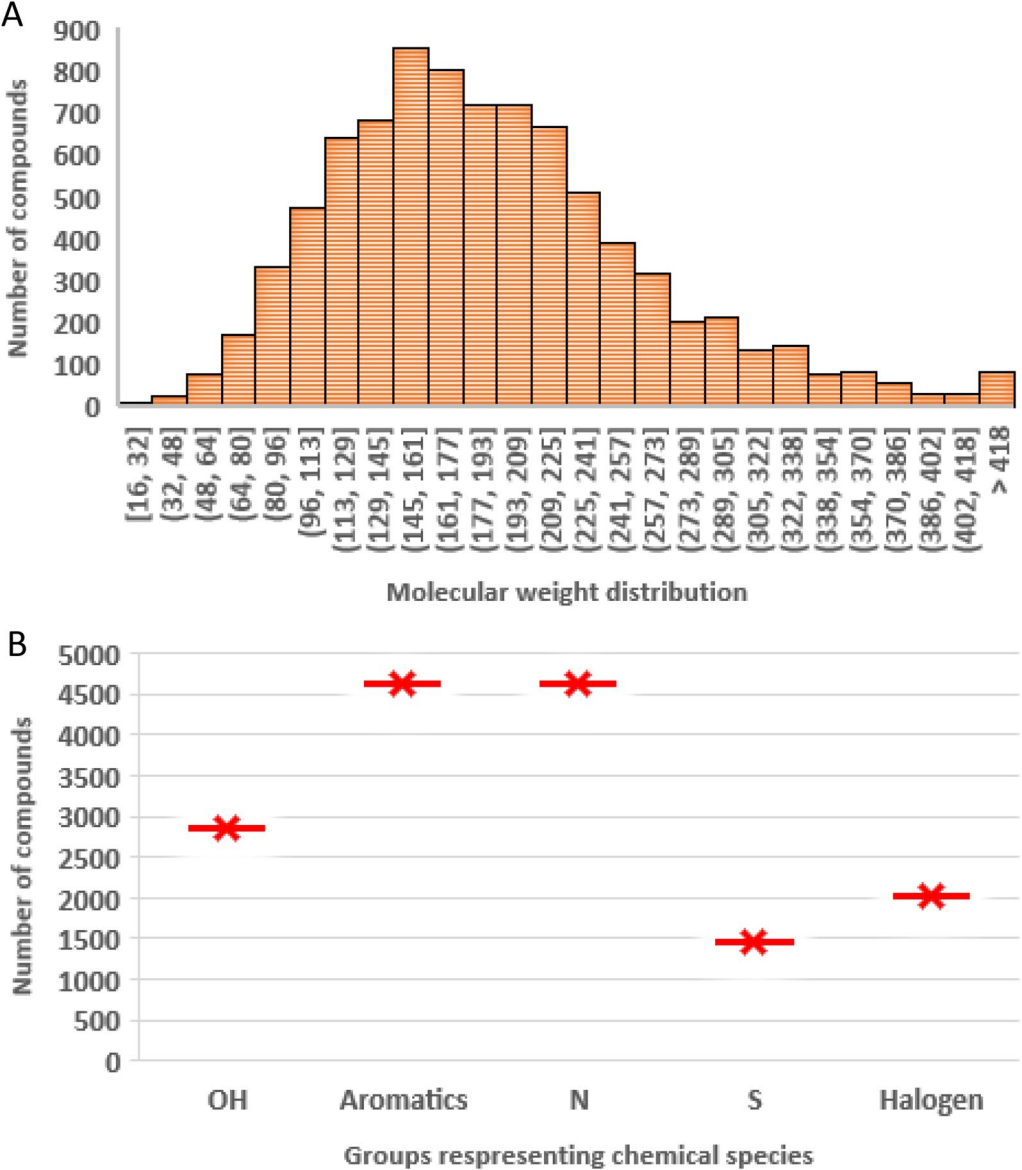
**A** molecular weight distribution of the produced dataset; **B** number of compounds in the dataset for each of the five nominated chemical species

Our database is a curated collection of the aqueous solubilities of organic compounds from three literature-based large databases: (1) Vermeire’s (11804 datapoints) [18], (2) Boobier’s (901 datapoints) [1], and (3) Delaney’s (1145 datapoints) [12]. The produced dataset was prepared by omitting the non-unique measures and noisy data, consisting of more than one solubility measure for a single molecule, yielding a total of 8,438 unique data entries (Additional file 1). The number of **C** (Carbon) atoms in each compound ranged from 1 to 12, representing the low molecular weight organic compounds with an average molecular weight of 190. They are of key interest due to their use as lead compounds in the search for new pharmacological effectors [19]. The range of molecular weights and the number of compounds containing N (Nitrogen), S (Sulfur), Halogens, OH, and aromatic groups is illustrated in Fig. 1.

A database of 100 reliable solubility measurements provided by Llinàs et al. [20] was selected for external validation. The set of molecules was disjointed from other data used in this study and was never used for model training or internal testing.

### Data preparation

We prepared the training data using the chemical and physical feature descriptor functions, i.e., molecular descriptors and circular fingerprint methods. Molecular descriptors define fragments as related physicochemical properties, or a collection of relevant structural features, such as a molecule’s ring count [2, 21]. A Descriptor-based model, which is considered as a standard ML approach, relies on a set of known descriptors [22]. We used Mordred package [23] to generate 1,613 two-dimensional (2D) descriptors and disregarded 3D descriptors to increase prediction speed and avoid repeatability problems regarding 3D descriptors values [24]. To prune the set of molecular descriptors, we initially excluded categorical variables, resulting in a reduced set of 811 descriptors out of the initial 1613. Subsequently, a correlation filter was applied using an optimized threshold of 0.1 to prune out the less relevant descriptors with low variance numeric, resulting in a selection of 506 descriptors. The descriptors’ pair correlation matrix was then calculated, and highly correlated descriptors were eliminated to prevent any particular mode of information from dominating the model’s mechanism. Furthermore, "FilterItLogS" feature was excluded from the descriptors to prevent data leakage from another ML model (FilterIt) that predicts solubility. This process yielded a final selection of 177 physicochemical descriptors.

Compared to molecular descriptors, fingerprinting methods provide a more dynamic representation that encompasses the characteristics of materials through their fragment features [25]. There are various types of molecular fingerprints, which are determined by the method used to convert the molecular fragment into a binary string [26]. Fingerprints with longer bit strings are more reliable for a similarity search since each significant bond in a molecule is defined separately as a sequence of binary digits (bits), and they have more stored information regarding the molecular properties. In this study, the Morgan algorithm was used as the circular fingerprinting method due to its exceptional performance in virtual screening experiments. This algorithm analyzes different fragments and encodes all possible molecular structure bonds [27]. Circular fingerprints are generated by considering the “circular” environment of each atom up to a given “radius” or “diameter” from the central atom [28]. The Morgan fingerprint, also known as extended-connectivity fingerprints (ECFPs), is the most popular circular fingerprint. This fingerprint perceives the presence of specific circular substructures around each atom in a molecule [29]. ECFPs is a method that identifies identical molecules with different atom numberings by representing the number of heavy-atom neighbors, number of hydrogen atoms, isotopes, and ring information. ECFPs are categorized into different types based on the selection of different maximum bond lengths or diameters of the circular atom neighborhood, where the digit at the end represents the maximum diameter value used to generate the fingerprint. We used a circular fingerprint with a diameter of four, ECFP4.

**Fig. 2:**
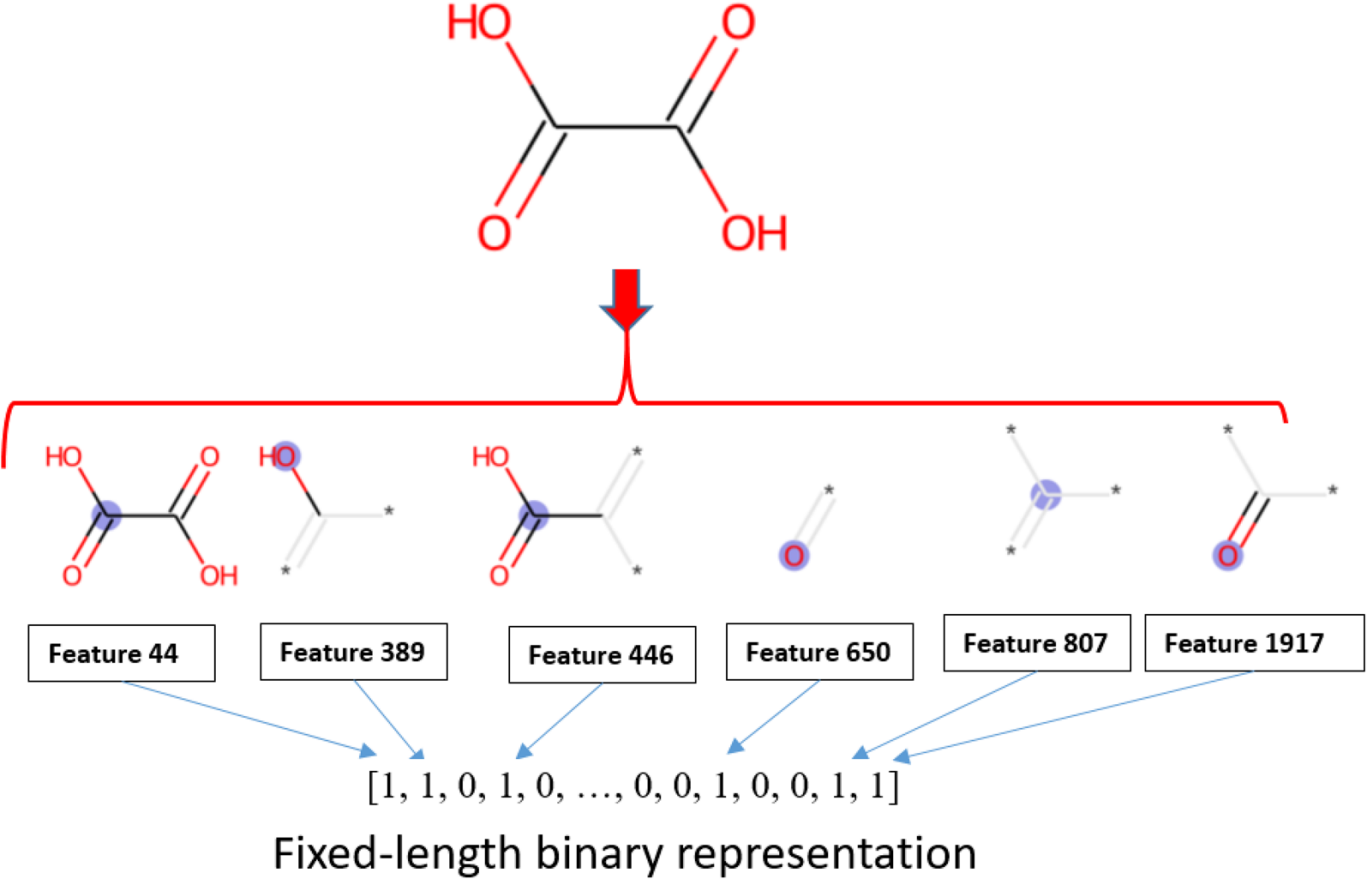
Molecular structure hashing to a list of bits using Morgan fingerprinting method

The schematic for transforming each molecular structure into a bit for Morgan fingerprints is illustrated in Fig. 2, where the path for transforming each molecular structure into a bit and the hashing technique are depicted. Chemical structures, as the SMILES form, were read by a machine and then hashed into a fingerprint with a size of 2,048 bits for all information bit-strings. Each bit was nominated as a single feature that can be used to survey the impact of various functional groups and their connectivity pathways on aqueous solubility [30].

### ML method

We randomly split our datasets into two groups: one for training and the other for testing the ML model and verifying the model’s accuracy. The training datasets comprised ~ 80% of our total database, representing approximately 6750 organic compounds. Random forest (RF) and Multiple Linear Regression (MLR) regressions were used in this study as our ML algorithms since they are among the most accurate general-purpose classifiers and also have fast computational efficiency[31, 32]. The training and test dataset sizes, RF hyperparameters, estimators, and random states were kept constant for all models during the analysis to improve comparisons.

ML models are treated as black boxes, and a model’s learning principles remain challenging; however, interpretations of a given feature’s impact on the prediction measures can still be obtained using SHAP (SHapley Additive exPlanations [33]) values. The effects of the most common physical quantities and characteristics of higher-performing features on aqueous solubility predictions were examined and the most important features for each method were obtained by performing SHAP analyses for all chemical representation methods implemented to structure the data into the ML model’s format. SHAP was chosen over other criteria, such as random forest feature or permutation importance, since it can be used to interpret complex model predictions. However, if a selected descriptor is a kind of a “vague” global value derived from the entire molecule, the interpretation becomes less clear. In order to overcome this limitation and gain a comprehensive understanding of the factors influencing solubility and the impact of diverse chemotypes on solubility, a sparse MLR coefficient approach was also employed. SHAP is based on the magnitude of feature attributions and assigns each feature an important value in comparison with permutation feature importance, which is based on the decrease in model performance. Additionally, SHAP values can be used to generate feature importance plots that show how each feature affects the model's output across the entire dataset. This provides a more comprehensive understanding of how the model is making predictions and offers a high level of interpretability for a model.

The mean absolute error (MAE) and root-mean-square deviation (RMSE) were used to approximate the models’ prediction accuracy and algorithm performance. In addition, we included the coefficient of determination (R^2), a frequently used statistical parameter. However, R^2 is not regarded as a reliable measure of model predictivity due to its sensitivity to model complexity and the number of parameters fitted in the model, in contrast to the MAE and RMSE metrics [34, 35].

## Results and discussion

### Chemical and physical feature descriptor methods

**Fig. 3:**
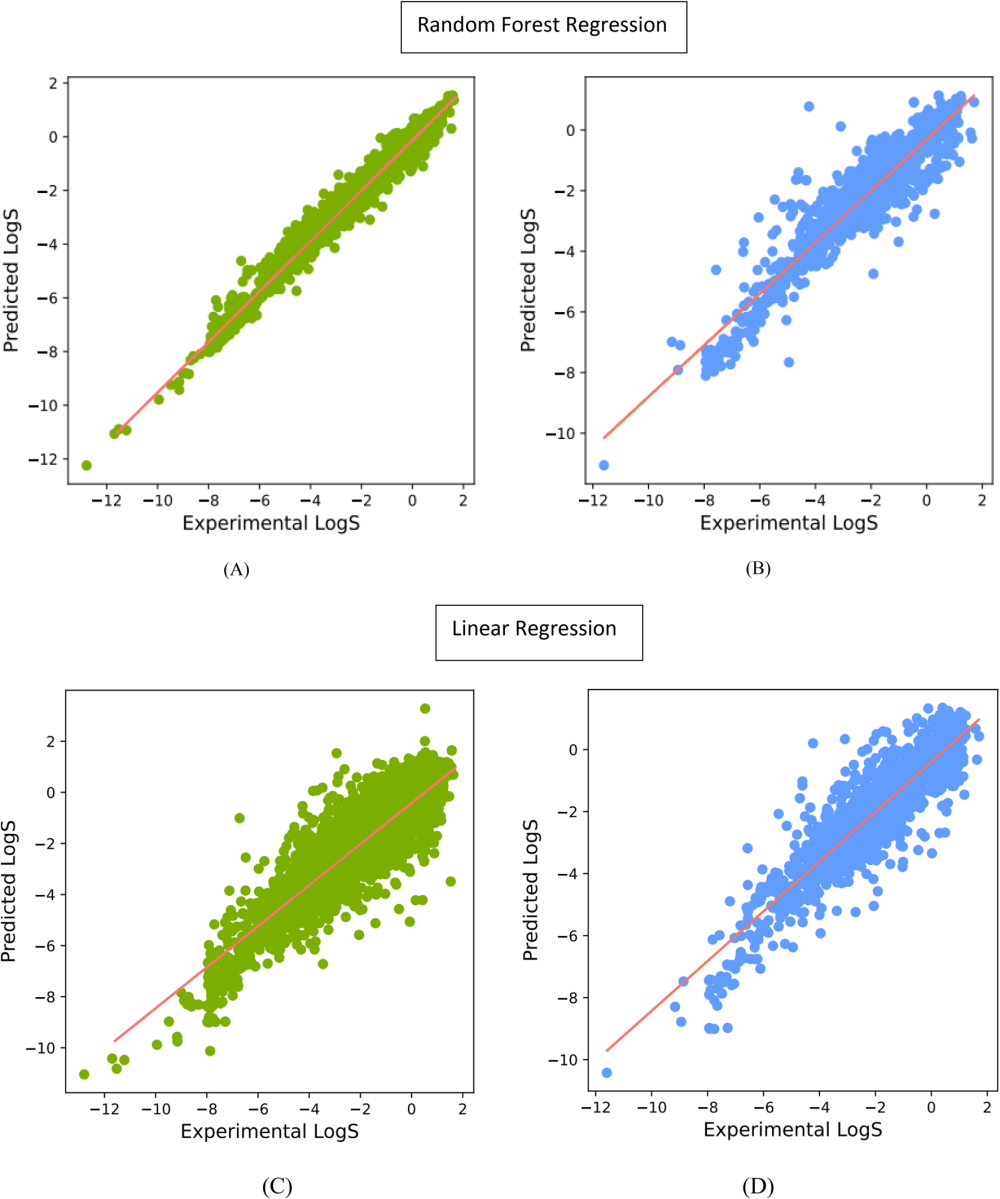
Estimated linear and Random Forest regressions for aqueous solubility predictions in the Molecular-descriptors method: **A** and **C** for training data; **B** and **D** for test data

The scatter plot in Fig. 3 demonstrates the relationship between the predicted LogS values derived from the Molecular-descriptors method and the corresponding measured values obtained from the RF and MLR models. The scatter plot encompasses data from both the training and test datasets. Significantly, the RF model demonstrates a stronger correlation and consequently delivers superior predictive performance when compared to the MLR model. This is evident from the higher R-squared (R^2) values of 0.88 and 0.80, as well as the lower RMSE and MAE values of 0.64/0.41 and 0.82/0.62, respectively, obtained for the test dataset. Table 2 provides a comprehensive summary of the accuracies associated with each of them.

**Table 2:** Estimated linear and Random Forest model evaluation for aqueous solubility predictions in the Molecular-descriptors method

	Training set			Test set		
	R^2^	RMSE	MAE	R^2^	RMSE	MAE
RF	0.98	0.25	0.16	0.88	0.64	0.41
MLR	0.80	0.82	0.61	0.80	0.82	0.62

In order to address the presence of data outliers, we employed the Local Outlier Factor (LOF) technique to identify and thoroughly examine outliers within the training dataset. The LOF model operates on a local level, assessing the degree of isolation of an object relative to its immediate neighborhood. This locality-based characteristic allows LOF to effectively detect outliers that may possess substantive significance but would remain undetectable using conventional approaches [36]. This approach ensured that only data points conforming to the normal distribution were retained, resulting in a more robust and reliable training dataset for subsequent analysis. The list of 177 physicochemical descriptors for outliers and inliers can be found in the GitHub repository associated with this work. T-test and corresponding P-values conducted on the physicochemical descriptors for outliers and inliers, revealed variations in the variable represented by ATSC2Z, ATSC2se, ATSC7Z, ATSC7i, EState_VSA4, NaaNH, PEOE_VSA3 and SlogP_VSA3 descriptors. Detailed information regarding the t-statistic and p-values for all 177 physicochemical descriptors can be found in Additional file 2: Table S1.

By removing outliers and recalculating the models, a slight improvement was observed in the MAE and RMSE values for test data in both the RF and MLR methods where the results can be found in Additional file 2: Table S2. Given that the predictions of the test set for models with and without outliers yielded essentially identical results, we present our analysis based on the complete data set, encompassing both outlier and non-outlier instances.

**Fig. 4:**
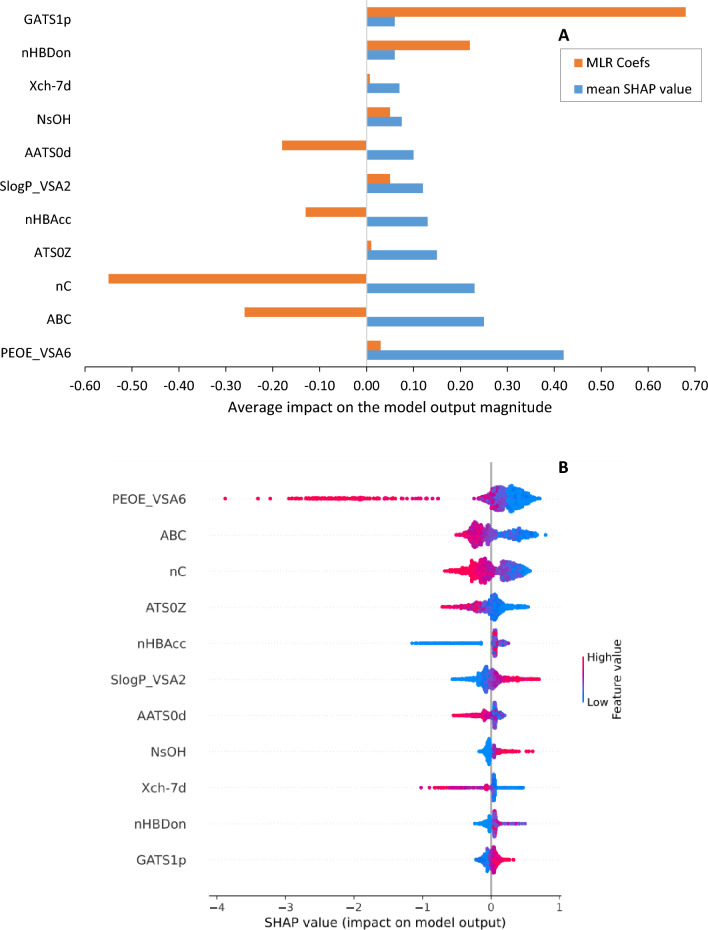
SHAP analysis of the RF model trained on the molecular descriptors: **A** average SHAP values and MLR Coefficients of each descriptor, and **B** impact of each descriptor on solubility output

Figure 4 illustrates the results of the SHAP analysis for the RF model trained on chemical descriptors and compares the impacts of top eleven chemical and physical descriptors, based on their average SHAP values, on the aqueous solubility outputs. In Fig. 4A, the blue bars depict the descriptors with the highest degree of impact, while Fig. 4B demonstrates the individual impact of each descriptor on the model's predictions. The feature values in the positive SHAP value range indicate a positive effect on solubility, while feature values in the negative SHAP value range indicate a negative effect. The density of the points represents the feature distribution. Red denotes a higher feature value, and blue denotes lower values.

**Fig. 5:**
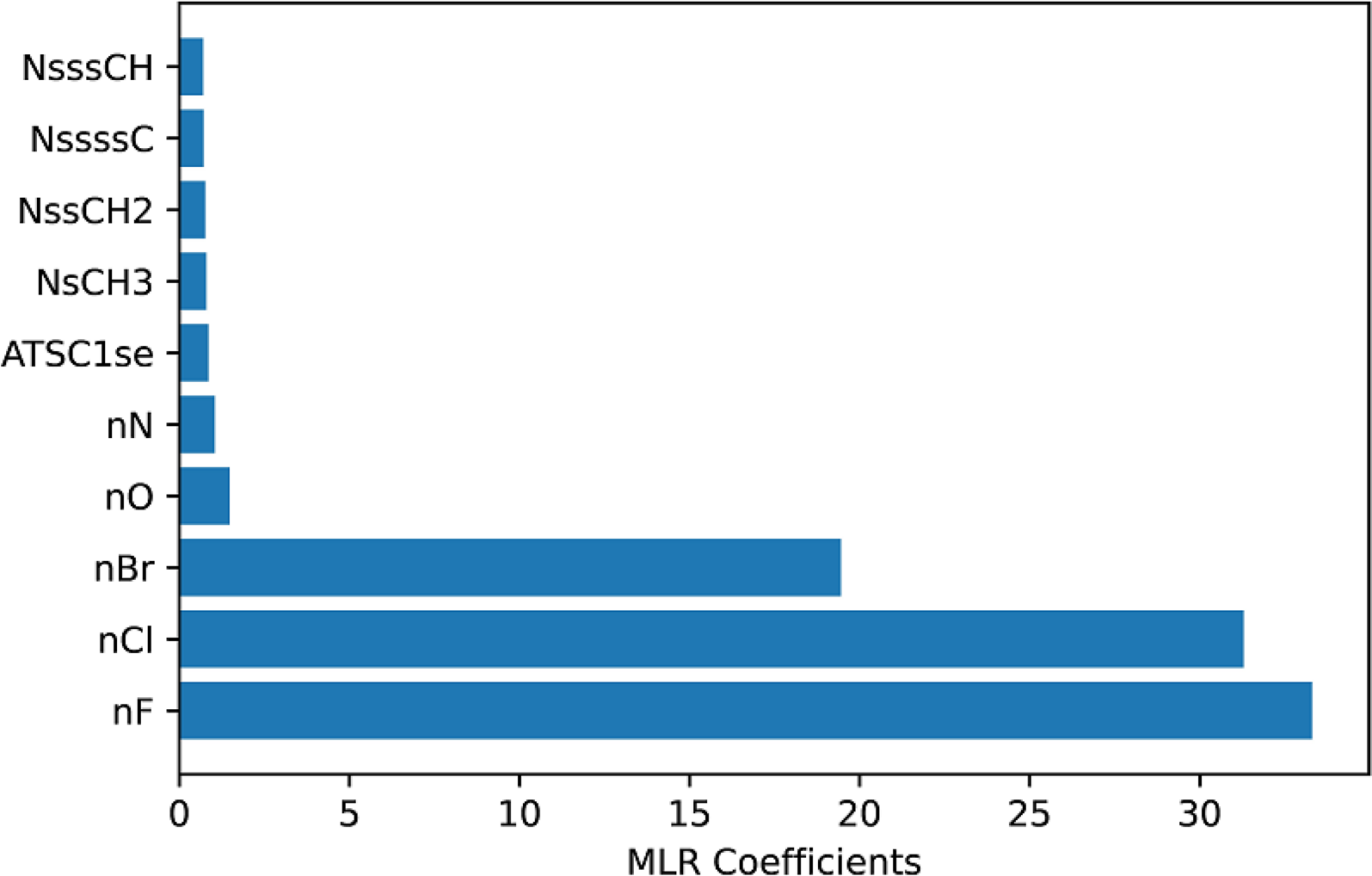
Top ten physiochemical descriptors with high MLR coefficient obtained from the MLR model trained on chemical descriptors. *nF* number of F atoms, *nCl* number of Cl atoms, *nBr* number of Br atoms, *nO* number of O atoms, *nN* number of N atoms, *ATSC1se* centered Moreau-Broto autocorrelation of lag 1 weighted by sanderson EN, *NsCH3* number of sCH3, *NssCH2* number of ssCH2, *NssssC* number of ssssC, *NsssCH* number of sssCH [37]

An MLR coefficient method was also utilized to identify the globally important chemical features, rather than the SHAP values, which exhibit local sensitivity. The MLR coefficient magnitudes corresponding to each of the top descriptors were incorporated into the Fig. 4, represented by the orange bars. Notably, the descriptors Slogp_VSA2, NsOH, NHBDon, and GATS1p, which exhibit positive SHAP values impact to the model's output, are characterized by higher MLR coefficient. As elucidated in Sect. “Fingerprinting methods”, feature importance in nonlinear models is a local rather than global property that depends on the location on the response surface where it is measured. Figure 5 illustrates the top ten physiochemical descriptors with high MLR coefficient obtained from the MLR model trained on chemical descriptors. Furthermore, Additional file 2: Table S1 provides a comprehensive list of all physiochemical descriptors along with their corresponding regression coefficients.

### Fingerprinting methods

**Fig. 6:**
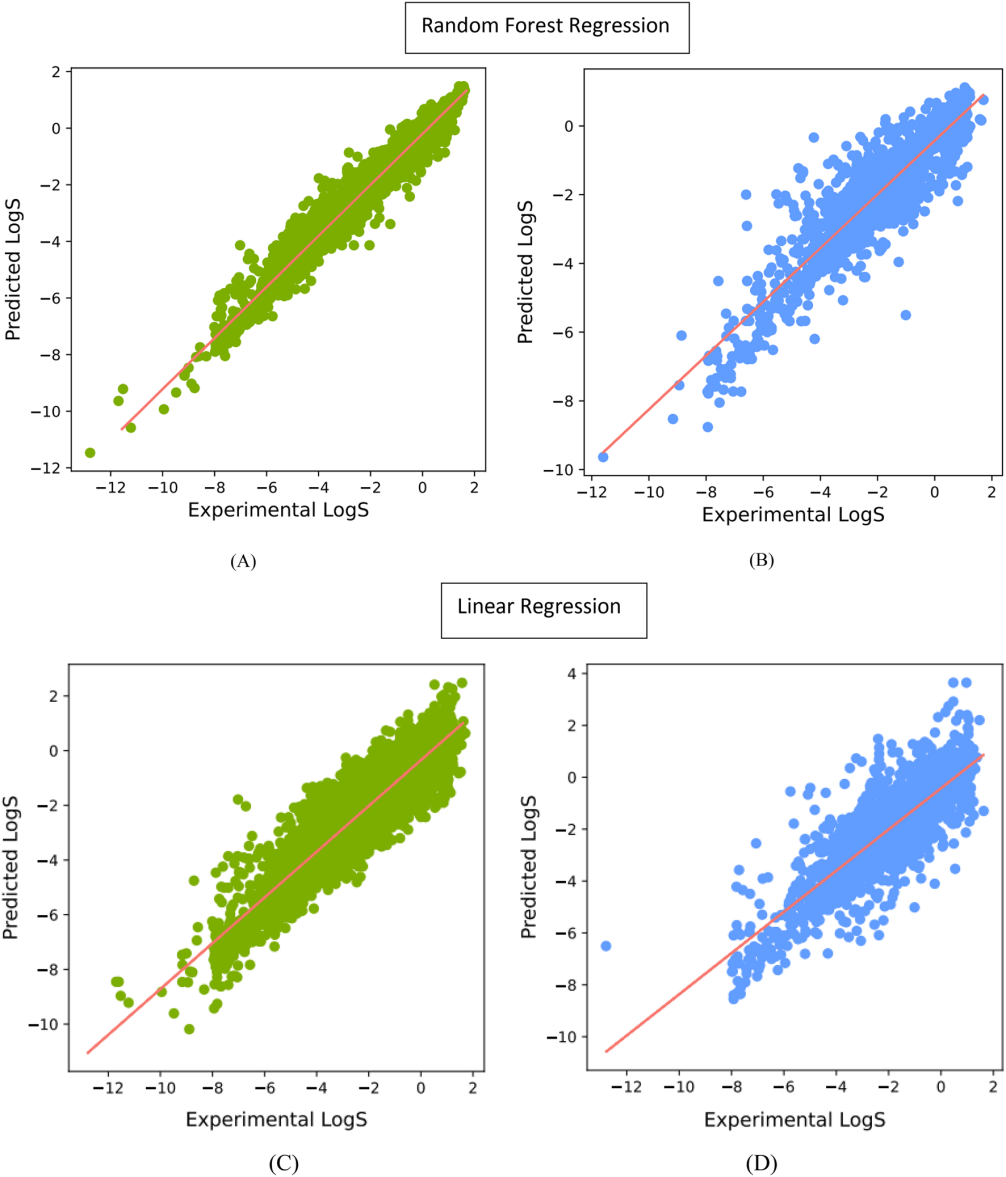
Performance of linear and Random Forest regressions for aqueous solubility predictions (**A**), (**C**) training data, and (**B**) and (**D**) test data; Morgan-Fingerprint method with 2048 features

To identify the most relevant features among the 2048 considered in the Morgan Fingerprint model, a feature selection technique using the LASSO model was employed. A range of alpha values, specifically 0.00001, 0.0001, 0.001, 0.01, 0.1, 1.0, and 10.0, were considered for optimization purposes. Subsequently, an alpha value of 0.001 was selected, resulting in the identification of 631 features from the original set. The performance of the pruned data was compared to the model with the 2048 features, as presented in Table 3. The findings indicate that when employing the RF model, the pruned data exhibited higher RMSE and MAE values. Conversely, for the MLR model, the pruned data demonstrated improved RMSE, MAE, and R^2 metrics. The improved results for MLR can be attributed to the inherent nature of LASSO, which acts as a regularization technique that performs variable selection and regularization by imposing a penalty on the absolute values of the regression coefficients. Figure 6 depicts the performance evaluation of the Morgan Fingerprint model with 2048 features using the RF and MLR algorithms.

**Table 3:** Estimated linear and Random Forest model evaluation for aqueous solubility predictions- Morgan-Fingerprint method

		Training set			Test set		
		R^2^	RMSE	MAE	R^2^	RMSE	MAE
2048 features	RF	0.96	0.35	0.23	0.81	0.80	0.55
	MLR	0.83	0.74	0.55	0.66	1.10	0.80
631 features	RF	0.96	0.35	0.23	0.81	0.84	0.57
	MLR	0.77	0.88	0.66	0.75	0.94	0.69

The Morgan fingerprints are binary representations that capture the presence or absence of specific structural features in molecules. In the context of Morgan fingerprints, which represent a sequence of bits rather than continuous variables, we utilized the Local Outlier Factor (LOF) technique to identify and scrutinize data outliers within the binary data. The LOF technique is applicable even with binary or categorical data, allowing us to detect and analyze potential outliers in the context of Morgan fingerprints' binary representations.

The RF and MLR models exhibited improved predictive performance upon excluding large outliers, although they demonstrated higher Absolute Calculation Error when applied to the blind dataset in Sect. “Blind test” (Additional file 2: Table S3). In order to investigate the nature of these outliers, an analysis was conducted on the frequency counts of each feature within the entire dataset. The findings revealed that some features appeared less than 100 times while some features appeared more than 3000 times. The ratio of “the number of features in an outlier’s fingerprint with less than 100 counts” to “the total number of features in outlier’s fingerprint” was calculated for all SMILES in each dataset. The results revealed that outliers exhibited a slightly higher mean ratio compared to inliers, with values of 0.31 and 0.29 respectively. This indicates that the frequency of each feature has a significant impact on the model's performance. Taking into account that removing outliers would result in a reduction in the occurrence of repeated features and considering the outcomes of the blind test, we made the decision to keep the model as-is without removing outliers. The complete list of outliers, inliers, and the aforementioned ratio has been uploaded to the associated GitHub repository for reference.

**Fig. 7:**
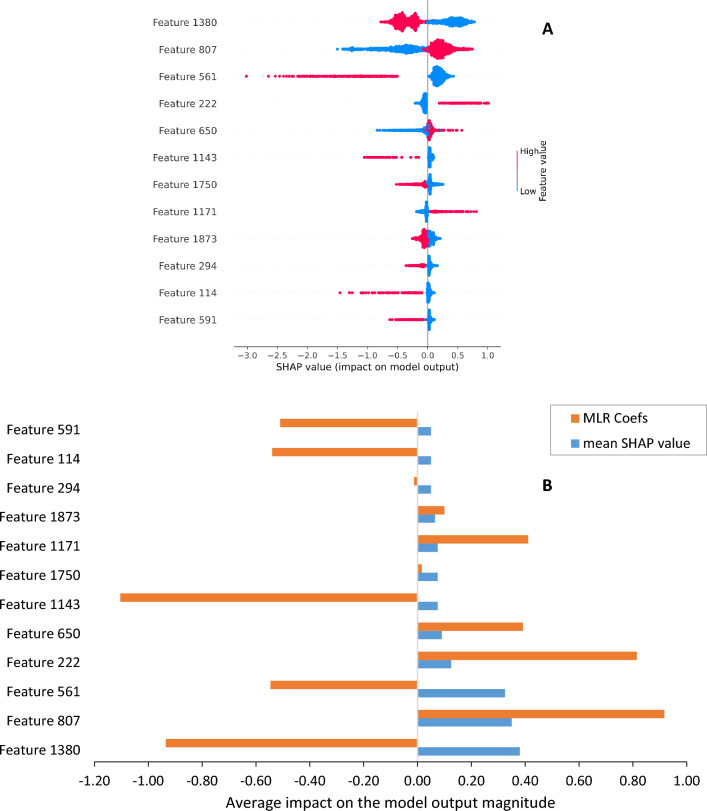
SHAP analysis of the ML model trained on the Morgan fingerprint **A** impact of each feature on solubility output, **B** average SHAP values and MLR Coefficients for the top twelve features

Figure 7 displays the top twelve important chemical substructures for predicting aqueous solubility based on their average SHAP values where the Morgan fingerprint was applied. Features 807, 222, 650, and 1171 are fragments consisting of the sp^2^ hybridized Carbon, hydroxyl group, carboxyl group and amine group respectively, contributed to increased solubility measures with enhanced amounts, and the rest negatively affected aqueous solubility. Solubility is a question of equilibria; therefore, to interpret the results we should frame the important features in terms of the energetics of the states as opposed to the dynamics that would transition between states. The energetics of a compounds in water can be estimated through a statistical thermodynamical-like approach [38, 39]. Gibbs energy, enthalpy or entropy as thermodynamic analysis of solubility with the purpose of contributing to the understanding of the possible molecular interactions can be used for interpretation of data [16]. Calculated Gibbs energy, as a thermodynamic paradigm, was adopted in this study to indicate a better mastery of the chemistry involved and improve the clarity of the discussion. Lower Gibbs energy measures indicate greater solubility in water, and a higher positive Gibbs energy specifies lower solubility in water. Table 4 illustrates the Gibbs energies for the top important features calculated by Perlovich’s equation (Eq. 1) [40].

1 $$\Delta {\text{G}}^{{{298}}} = \, \left( { - 0.{5} \pm {1}.{6}} \right) \, {-} \, \left( {{1}.{37} \pm 0.0{6}} \right)\alpha \, + \, \left( {{3}.{84} \pm 0.{25}} \right)\sum {\text{Ca }}{-} \, \left( {{2}.{97} \pm 0.{26}} \right)\sum {\text{Cd}}$$

where α is molecular polarizability, ƩCa is the sum of all H-bond acceptor factors in a molecule, and ƩCd is the sum of H-bond donor factors. Three described variables for each feature were calculated by descriptors-based method described in Sect. "Chemical and physical feature descriptor methods".

**Table 4:** Gibbs energies and MLR Coefficients for the top twelve features

Substructure	Substructure drawings	Molecular polarizability	H-bond acceptor	H-bond Donor	∆G^298^	MLR. coefficients
Feature 1380		12.01	0	0	14.46	−0.93
Feature 807		1.47	0	1	0.29	0.91
Feature 561		10.52	0	0	12.42	−0.54
Feature 222		4.47	1	1	−2.99	0.81
Feature 650		0.80	0	0	−0.90	0.39
Feature 1143		15.02	0	0	18.57	−1.10
Feature 1750		16.69	0	0	20.86	0.01
Feature 1171		2.43	0	0	1.33	0.41
Feature 1873		9.01	0	0	10.34	0.10
Feature 294		17.10	4	0	7.14	−0.013
Feature 114		16.35	0	0	20.40	−0.54
Feature 591		12.68	0	0	15.37	−0.51

Features 807, 222, 650, and 1171, as the features with positive effects, have low Gibbs energies and are thermodynamically favorable; they have lower Gibbs energies compared to Features 1380, 561, 1143, 1750, 114 and 591 with negative effects. The thermodynamic results are intuitive and agree with expectations arising from SHAP’s analysis. The agreement between the impactful features and the thermodynamic quantities can separated the fingerprint method from other computational tools to predict the physico-chemical properties [41].

**Fig. 8:**
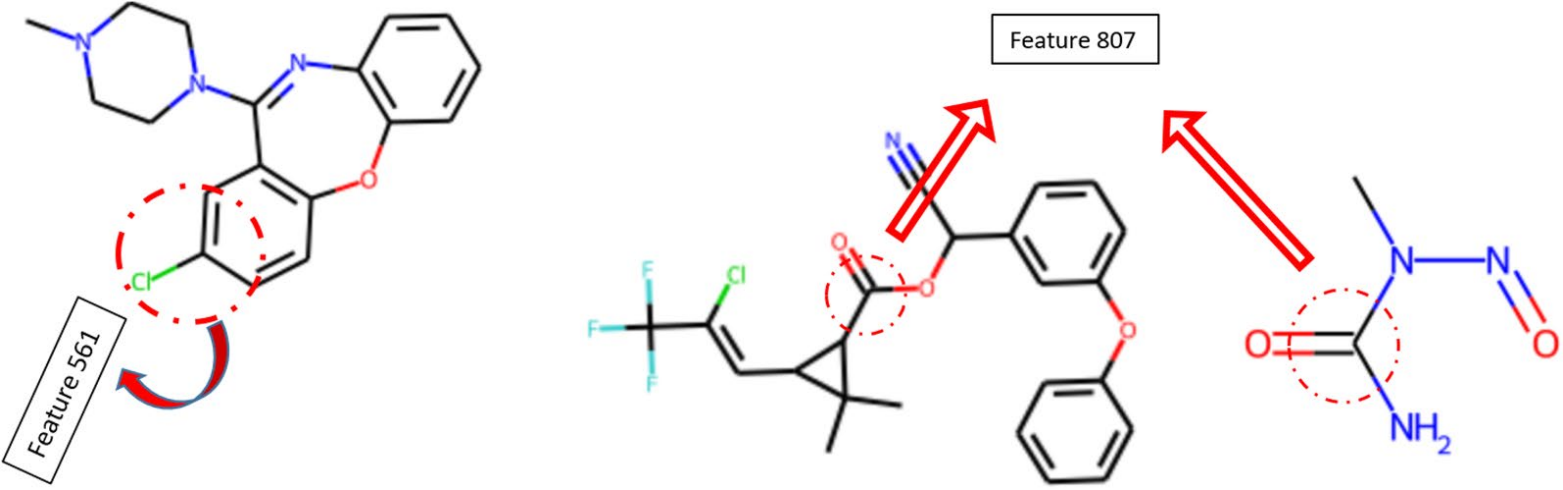
Illustrations of the structure positions of the three selected features extracted by Morgan fingerprint

Blue represents the central atom, yellow depicts the aromatic atoms, and the aliphatic ring atoms are highlighted in dark gray in the substructure drawings illustrated in Table 4. Light gray also indicates atom/bond structures that influence the atom’s connectivity invariants but are not directly part of the fingerprint. A schematic of extracting features 561 and 807 from their molecular structure is provided in Fig. 8 to illustrate the concept of hashing each structure.

To have a robust illustration of the factors driving solubility and the role of diverse chemotype on solubility, a sparse MLR coefficient approach was also utilized. Feature importance in nonlinear models is a local rather than global property that depends on the location on the response surface where it is measured. This is evident in the SHAP graphs that span ranges from negative to positive influences on the model rather than having a single value. Given that the MLR model has a better RMSE than the RF model, the regression coefficients of the MLR model were analyzed to gain insight into how different features modulate solubility in linear model. The last column of Table 4 summarizes the regression coefficient corresponding to each of the twelve important features. Additionally, the MLR coefficients magnitudes for each important feature were added to Fig. 7 as the orange bars to facilitate a comparison between the nonlinear feature importances obtained from SHAP (blue bars) and the linear MLR coefficients. Notably, high measures of the regression coefficient for features 807, 222, 650, and 1171, as well as the low measures for features 1380, 561, 1143, 1750, 114 and 591, align with the expectations arising from SHAP’s analysis for the RF model and Gibbs energy results. Additional file 2: Table S4 provides a list of the top 50 features with positive regression coefficients, further elucidating the role of different features in modulating solubility.

### Blind test

We performed a blind test on a database that was never used in our model to verify performance and compare the two models. The database consists of 32 low molecular weight organic molecules with the number of C atoms ranging from 1 to 12, extracted from the dataset of 100 druglike molecules at 25 °C from the Llinàs et al. [20] study. It is worth mentioning that identifying a reliable benchmark reference for solubility can be challenging due to the multiple definitions that exist, and ambiguity in reported values. Furthermore, the medium used for measurements, such as distilled or pH-buffered water, can yield significantly different results. We selected this dataset as the benchmark reference since it reports the intrinsic solubility. This parameter refers to the solubility of a compound in its free acid or free base form, which is independent of the medium’s pH and it is rather more reproducible than other measures. Thus, the selection of intrinsic solubility as our benchmark reference allows for a more standardized and reliable comparison of solubility values, and contribute to the accuracy and precision of our research findings.

Table 5 displays the performance of the random forest (RF) model in predicting the aqueous solubility of the benchmark dataset using two distinct methods, namely the Morgan fingerprint (MF) and physicochemical descriptors. The results show MF model outperformed the physicochemical model in predicting the blind set, whereas the latter achieved higher accuracy on the test set (RMSE 0.80 versus 0.64). To address this anomalous discrepancy, it should be noted that the performance of an ML model on a test set may not necessarily predict its performance on a blind set. The test set and the blind set may differ in ways that affect the predictive accuracy of the models, such as the types of compounds, the chemical properties, and the experimental conditions. To further assess the robustness of the models, we tested our models using a different dataset comprised of also 32 compounds that were listed in the “Solubility Challenge” section of the Llinàs study [20]. The results of this challenge test are summarized in Additional file 2: Table S5. The mean averages of the estimated error are 0.64 and 1.12 logS for MF and MD models, respectively. The performance of the MF model reflects its potential usefulness in predicting the solubility of drug-like molecules. Interestingly, our MF model was trained on low molecular weight molecules (an average molecular weight around 190) with the number of C atoms ranging from 1 to 12, whereas the 32 drug-like molecules in this challenge had a significantly higher molecular weight and more carbon atoms (an average of 296 molecular weight and 19 carbon atoms).

**Table 5:** Empirical and predicted solubility for selected druglike molecules using different chemical representation methods

Name	logS(mol/L): Intrinsic Solubility	logS(mol/L): Molecular Descriptor Method	Molecular Descriptor Method -Absolute Calculation Error	logS(mol/L): MF^1^ Method	MF Method-Absolute Calculation Error
Hexobarbital	−2.67	−1.69	0.98	−2.40	0.27
Nalidixic_acid	−3.61	−1.54	2.07	−3.43	0.18
Phenanthroline	−1.61	−1.93	0.32	−1.80	0.19
Phenobarbital	−2.29	−2.14	0.15	−2.33	0.04
Sulfamethazine	−2.73	−1.56	1.17	−2.38	0.35
Bromogramine	−4.05	−1.68	2.37	−3.92	0.13
Phenazopyridine	−4.19	−2.08	2.11	−4.02	0.17
Amantadine	−1.85	−1.89	0.04	−2.12	0.27
Benzylimidazole	−2.25	−1.66	0.59	−1.51	0.75
Chlorpropamide	−3.24	−1.67	1.57	−2.89	0.35
Cimetidine	−1.69	−1.86	0.17	−1.49	0.20
Thymol	−2.18	−1.90	0.28	−2.26	0.08
Tryptamine	−3.29	−1.90	1.39	−2.91	0.39
Azathioprine	−3.2	−1.76	1.44	−2.84	0.36
Sulfathiazole	−2.68	−1.41	1.27	−2.55	0.13
Acetaminophen	−1.06	−1.50	0.44	−1.19	0.13
Diazoxide	−3.36	−1.88	1.48	−3.28	0.09
Famotidine	−2.64	−1.77	0.87	−2.58	0.06
Hydroflumethiazide	−2.96	−1.93	1.03	−2.33	0.63
Nitrofurantoin	−3.23	−1.96	1.27	−3.42	0.19
Phthalic_acid_form_I	−1.49	−1.86	0.37	−0.93	0.56
Sulfacetamide	−1.51	−1.64	0.13	−1.42	0.09
Trichloromethiazide_ Form_I	−3.18	−2.11	1.07	−2.78	0.40
2_amino_5_ Bromobenzoic_acid	−3.07	−1.57	1.50	−2.80	0.27
5_bromo_2_4_ Dihydroxybenzoic_acid	−2.62	−2.74	0.12	−2.20	0.42
Chlorzoxazone	−2.65	−2.07	0.58	−2.89	0.24
5_hydroxybenzoic_acid	−1.46	−1.31	0.15	−1.69	0.23
4_iodophenol	−1.71	−1.70	0.01	−2.00	0.29
Metronidazole	−1.22	−1.55	0.33	−1.35	0.13
Guanine	−4.42	−1.92	2.50	−4.08	0.34
Acetazolamide	−2.43	−1.69	0.74	−2.34	0.09
1_naphthol	−1.98	−1.89	0.09	−2.27	0.29
^1^Morgan Fingerprint			Mean = 0.89		Mean = 0.25

The results in Table 5 and Additional file 2: Table S5 indicate an acceptable difference since the average uncertainty in measured aqueous solubility for organic molecules typically ranges from ∼0.6 to one order of magnitude, as reported in previous studies [20, 42–44]. The reason behind this can be attributed to the fact that the reported solubility values were gathered from various published works under varied experimental conditions. Furthermore, differences in solubility between different polymorphs of a given substance can also contribute to the mean average error of the models. Additionally, there may be confusion in identifying the type of solubility reported, as intrinsic solubilities can be mistakenly assumed to be thermodynamic values or kinetic measures. It is important to differentiate between these concepts: kinetic solubility refers to the dissolution rate of a substance, while thermodynamic solubility represents the equilibrium concentration of the solute in the solvent. In contrast, intrinsic solubility pertains to the solubility of a compound in its free acid or free base form. The kinetic solubility cannot be used as a reliable guide to the intrinsic or thermodynamic solubility of a compound, given its strong dependence on time and experimental parameters [45]. Stuart et al. highlighted a significant difference between the kinetic approximation of solubility and the intrinsic solubility of some compounds [45]. For instance, diclofenac exhibited precipitation levels that surpassed 50 times its intrinsic solubility. Similarly, Saal et al. investigated the differences between thermodynamic and kinetic solubility [46]. They reported mean differences of 0.22 log units and maximum differences of 1.96 log units for compounds where the residue of the thermodynamic assay exhibited a crystalline nature. Conversely, for compounds with an amorphous residue, the mean differences were 0.04 log units, with maximum differences reaching 0.89 log units.

## Conclusions

We compared two supervised machine learning implementations to predict the aqueous solubility of various components using two distinct cheminformatics methods. We used molecular descriptors and fingerprints as the chemical representation methods. Our results were compared to a blind, low molecular database with specified aqueous solubility experiments, revealing that using a fingerprint method has a lower average absolute calculation error, which is comparable to other group contribution methods currently available. We also gained insight into how important features impact an ML’s output using SHAP analysis and calculated Gibbs energies for these features to investigate their thermodynamic favorability. Compare to the fingerprint model, the physicochemical descriptor model has demonstrated better predictive accuracy for the given test set and can incorporate more complex information.

### Supplementary Information

**Additional file 1.** Dataset.

**Additional file 2: Table S1**. List of descriptors along with their corresponding MLR coefficients, T-statistics and P-values used to predict aqueous solubility. **Table S2**. Evaluation of estimated Linear and Random Forest models for aqueous solubility predictions with outliers removed using the Molecular-Descriptors Method. **Table S3**. Evaluation of estimated Linear and Random Forest models for aqueous solubility predictions with outliers removed using the Morgan-Fingerprint method with key 2048 features. **Table S4**. List of the top 50 features in Morgan-Fingerprint with positive regression coefficients in predicting the aqueous solubility. **Table S5**. Performance of Molecular-Descriptors and Morgan-Fingerprint Methods in Predicting the Aqueous Solubility for 32 Compounds in the “Solubility Challenge”.

## Data Availability

The code and datasets for our ML implementation can be found at: https://github.com/arashtayyebi/prediction-of-water-solubility.git.
