# Supplementary material for: Prediction of organic compound aqueous solubility using machine learning: a comparison study of descriptor-based and fingerprints-based models
Source: J Cheminform. 2023 Oct 18;15:99. doi: 10.1186/s13321-023-00752-6 (PMC10583449; doi:10.1186/s13321-023-00752-6)
Supplement: Supplementary file 2 — Additional file 2: Table S1. List of descriptors along with their corresponding MLR coefficients, T-statistics and P-values used to predict aqueous solubility. Table S2. Evaluation of estimated Linear and Random Forest models for aqueous solubility predictions with outliers removed using the Molecular-Descriptors Method. Table S3. Evaluation of estimated Linear and Random Forest models for aqueous solubility predictions with outliers removed using the Morgan-Fingerprint method with key 2048 features. Table S4. List of the top 50 features in Morgan-Fingerprint with positive regression coefficients in predicting the aqueous solubility. Table S5. Performance of Molecular-Descriptors and Morgan-Fingerprint Methods in Predicting the Aqueous Solubility for 32 Compounds in the “Solubility Challenge”. [file 13321_2023_752_MOESM2_ESM.docx]

# Additional file

**Prediction of Organic Compound Aqueous Solubility Using Machine Learning- A Comparison Study of Descriptor-Based and Fingerprints-Based Models**

**Arash Tayyebi^a^, Ali S.Alshami^a*^, Zeinab Rabiei**^b^**, Xue Yu^c^, Nadhem Ismail^a^, Musabbir Jahan Talukder**^a^**, , Jason Power^d^**

***Corresponding author: ali.alshami@und.edu, Tel: +1 701 777 6838**

**a University of North Dakota, Chemical Engineering, Grand Forks, ND, 58201, USA**

**b University of North Dakota, Chemistry Department, Grand Forks, ND, 58202, USA**

**c Energy & Environmental Research Center, University of North Dakota, Grand Forks, ND, 58202, USA**

**d University of North Dakota, Biomedical Sciences, Grand Forks, ND, 58202, USA**

**Table S1. List of descriptors along with their corresponding MLR coefficients, T-statistics and P-values used to predict aqueous solubility**

| **Name** | **MLR Coefficient** | **Description** | **T-statistic** | **P-value** |
| --- | --- | --- | --- | --- |
| AATS0d | -0.18 | averaged moreau-broto autocorrelation of lag 0 weighted by sigma electrons | -0.54 | 0.59 |
| AATS0dv | -0.06 | averaged moreau-broto autocorrelation of lag 0 weighted by valence electrons | -0.30 | 0.77 |
| AATS0i | 0.00 | averaged moreau-broto autocorrelation of lag 0 weighted by ionization potential | 0.42 | 0.67 |
| AATSC0dv | -0.07 | averaged and centered moreau-broto autocorrelation of lag 0 weighted by valence electrons | -0.83 | 0.40 |
| AATSC0i | 0.01 | averaged and centered moreau-broto autocorrelation of lag 0 weighted by ionization potential | -0.32 | 0.75 |
| AATSC0v | -0.01 | averaged and centered moreau-broto autocorrelation of lag 0 weighted by vdw volume | -0.58 | 0.56 |
| AATSC1Z | -0.01 | averaged and centered moreau-broto autocorrelation of lag 1 weighted by atomic number | -1.22 | 0.22 |
| ABC | -0.26 | atom-bond connectivity index | -1.49 | 0.14 |
| ATS0Z | 0.01 | moreau-broto autocorrelation of lag 0 weighted by atomic number | -0.53 | 0.60 |
| ATSC0i | 0.02 | centered moreau-broto autocorrelation of lag 0 weighted by ionization potential | -1.55 | 0.12 |
| ATSC1d | -0.02 | centered moreau-broto autocorrelation of lag 1 weighted by sigma electrons | -0.96 | 0.34 |
| ATSC1dv | 0.02 | centered moreau-broto autocorrelation of lag 1 weighted by valence electrons | 0.57 | 0.57 |
| ATSC1i | -0.01 | centered moreau-broto autocorrelation of lag 1 weighted by ionization potential | 0.79 | 0.43 |
| ATSC1p | -0.17 | centered moreau-broto autocorrelation of lag 1 weighted by polarizability | 1.47 | 0.14 |
| ATSC1se | 0.87 | centered Moreau-Broto autocorrelation of lag 1 weighted by sanderson EN | 0.98 | 0.33 |
| ATSC1v | 0.01 | centered moreau-broto autocorrelation of lag 1 weighted by vdw volume | -0.49 | 0.63 |
| ATSC1Z | 0.00 | centered moreau-broto autocorrelation of lag 1 weighted by atomic number | -1.53 | 0.13 |
| ATSC2d | 0.11 | centered moreau-broto autocorrelation of lag 2 weighted by sigma electrons | 0.44 | 0.66 |
| ATSC2dv | -0.01 | centered moreau-broto autocorrelation of lag 2 weighted by valence electrons | -1.06 | 0.29 |
| ATSC2i | 0.00 | centered moreau-broto autocorrelation of lag 2 weighted by ionization potential | -1.31 | 0.19 |
| ATSC2se | 0.08 | centered moreau-broto autocorrelation of lag 2 weighted by sanderson EN | -2.00 | 0.05 |
| ATSC2v | 0.00 | centered moreau-broto autocorrelation of lag 2 weighted by vdw volume | -1.50 | 0.13 |
| ATSC2Z | 0.00 | centered moreau-broto autocorrelation of lag 2 weighted by atomic number | -2.28 | 0.02 |
| ATSC3d | 0.04 | centered moreau-broto autocorrelation of lag 3 weighted by sigma electrons | 0.04 | 0.96 |
| ATSC3dv | -0.01 | centered moreau-broto autocorrelation of lag 3 weighted by valence electrons | 0.78 | 0.43 |
| ATSC3i | 0.00 | centered moreau-broto autocorrelation of lag 3 weighted by ionization potential | -0.97 | 0.33 |
| ATSC3p | -0.03 | centered moreau-broto autocorrelation of lag 3 weighted by polarizability | 1.27 | 0.20 |
| ATSC3se | 0.13 | centered moreau-broto autocorrelation of lag 3 weighted by sanderson EN | 0.62 | 0.54 |
| ATSC3v | 0.00 | centered moreau-broto autocorrelation of lag 3 weighted by vdw volume | 1.44 | 0.15 |
| ATSC3Z | 0.00 | centered moreau-broto autocorrelation of lag 3 weighted by atomic number | 0.77 | 0.44 |
| ATSC4d | 0.05 | centered moreau-broto autocorrelation of lag 4 weighted by sigma electrons | 0.69 | 0.49 |
| ATSC4dv | 0.00 | centered moreau-broto autocorrelation of lag 4 weighted by valence electrons | -1.88 | 0.06 |
| ATSC4i | -0.01 | centered moreau-broto autocorrelation of lag 4 weighted by ionization potential | 0.27 | 0.79 |
| ATSC4p | 0.00 | centered moreau-broto autocorrelation of lag 4 weighted by polarizability | -0.58 | 0.56 |
| ATSC4se | 0.03 | centered moreau-broto autocorrelation of lag 4 weighted by sanderson EN | -0.22 | 0.82 |
| ATSC4v | 0.00 | centered moreau-broto autocorrelation of lag 4 weighted by vdw volume | -0.05 | 0.96 |
| ATSC4Z | 0.00 | centered moreau-broto autocorrelation of lag 4 weighted by atomic number | -0.39 | 0.70 |
| ATSC5d | 0.04 | centered moreau-broto autocorrelation of lag 5 weighted by sigma electrons | 0.14 | 0.89 |
| ATSC5dv | 0.00 | centered moreau-broto autocorrelation of lag 5 weighted by valence electrons | -0.93 | 0.35 |
| ATSC5i | -0.01 | centered moreau-broto autocorrelation of lag 5 weighted by ionization potential | 1.43 | 0.15 |
| ATSC5p | 0.02 | centered moreau-broto autocorrelation of lag 5 weighted by polarizability | 0.53 | 0.59 |
| ATSC5se | 0.03 | centered moreau-broto autocorrelation of lag 5 weighted by sanderson EN | -1.29 | 0.20 |
| ATSC5v | 0.00 | centered moreau-broto autocorrelation of lag 5 weighted by vdw volume | -0.87 | 0.38 |
| ATSC5Z | 0.00 | centered moreau-broto autocorrelation of lag 5 weighted by atomic number | -1.13 | 0.26 |
| ATSC6d | 0.03 | centered moreau-broto autocorrelation of lag 6 weighted by sigma electrons | 1.22 | 0.22 |
| ATSC6dv | 0.00 | centered moreau-broto autocorrelation of lag 6 weighted by valence electrons | 0.85 | 0.39 |
| ATSC6i | -0.01 | centered moreau-broto autocorrelation of lag 6 weighted by ionization potential | 1.51 | 0.13 |
| ATSC6se | 0.12 | centered Moreau-Broto autocorrelation of lag 6 weighted by sanderson EN | 0.71 | 0.47 |
| ATSC6v | 0.00 | centered moreau-broto autocorrelation of lag 6 weighted by vdw volume | 1.14 | 0.25 |
| ATSC6Z | 0.00 | centered moreau-broto autocorrelation of lag 6 weighted by atomic number | 1.96 | 0.05 |
| ATSC7d | 0.01 | centered moreau-broto autocorrelation of lag 7 weighted by sigma electrons | 0.80 | 0.42 |
| ATSC7dv | 0.00 | centered moreau-broto autocorrelation of lag 7 weighted by valence electrons | 1.39 | 0.17 |
| ATSC7i | 0.00 | centered moreau-broto autocorrelation of lag 7 weighted by ionization potential | 1.97 | 0.05 |
| ATSC7p | -0.02 | centered moreau-broto autocorrelation of lag 7 weighted by polarizability | 1.56 | 0.12 |
| ATSC7v | 0.00 | centered moreau-broto autocorrelation of lag 7 weighted by vdw volume | 1.61 | 0.11 |
| ATSC7Z | 0.00 | centered moreau-broto autocorrelation of lag 7 weighted by atomic number | 2.58 | 0.01 |
| ATSC8d | 0.02 | centered moreau-broto autocorrelation of lag 8 weighted by sigma electrons | -0.64 | 0.52 |
| ATSC8dv | 0.00 | centered moreau-broto autocorrelation of lag 8 weighted by valence electrons | 1.57 | 0.12 |
| ATSC8i | 0.00 | centered moreau-broto autocorrelation of lag 8 weighted by ionization potential | -1.59 | 0.11 |
| ATSC8v | 0.00 | centered moreau-broto autocorrelation of lag 8 weighted by vdw volume | -0.28 | 0.78 |
| ATSC8Z | 0.00 | centered moreau-broto autocorrelation of lag 8 weighted by atomic number | 1.20 | 0.23 |
| BalabanJ | 0.41 | Balaban’s J index | -0.68 | 0.50 |
| C1SP2 | -0.04 | SP2 carbon bound to 1 other carbon | -1.88 | 0.06 |
| C1SP3 | -0.02 | SP3 carbon bound to 1 other carbon | -0.77 | 0.44 |
| C2SP3 | -0.16 | SP3 carbon bound to 2 other carbons | 0.21 | 0.84 |
| C3SP2 | -0.11 | SP2 carbon bound to 3 other carbons | -1.75 | 0.08 |
| C3SP3 | -0.06 | SP3 carbon bound to 3 other carbons | -1.72 | 0.09 |
| CIC1 | -0.84 | 1-ordered complementary information content | 0.03 | 0.98 |
| Diameter | 0.00 | topological diameter | 0.56 | 0.57 |
| ECIndex | 0.00 | eccentric connectivity index | 0.12 | 0.91 |
| EState_VSA1 | 0.00 | EState VSA Descriptor 1 (-inf < x < -0.39) | -0.99 | 0.32 |
| EState_VSA2 | 0.02 | EState VSA Descriptor 2 ( -0.39 <= x < 0.29) | -1.09 | 0.27 |
| EState_VSA3 | 0.02 | EState VSA Descriptor 3 ( 0.29 <= x < 0.72) | 1.33 | 0.18 |
| EState_VSA4 | 0.03 | EState VSA Descriptor 4 ( 0.72 <= x < 1.17) | -2.28 | 0.02 |
| EState_VSA5 | 0.01 | EState VSA Descriptor 5 ( 1.17 <= x < 1.54) | -0.62 | 0.54 |
| EState_VSA6 | 0.03 | EState VSA Descriptor 6 ( 1.54 <= x < 1.81) | 0.32 | 0.75 |
| EState_VSA7 | 0.03 | EState VSA Descriptor 7 ( 1.81 <= x < 2.05) | 0.44 | 0.66 |
| EState_VSA8 | 0.01 | EState VSA Descriptor 8 ( 2.05 <= x < 4.69) | -0.92 | 0.36 |
| GATS1i | -0.30 | GATS1i | 0.28 | 0.78 |
| GATS1p | 0.69 | geary coefficient of lag 1 weighted by polarizability | -0.70 | 0.48 |
| IC1 | -0.38 | 1-ordered neighborhood information content | -1.67 | 0.09 |
| n5ARing | -0.08 | 5-membered aromatic ring count | -0.89 | 0.37 |
| n5aRing | -0.05 | 5-membered aromatic ring count | -0.28 | 0.78 |
| n5HRing | 0.09 | 5-membered hetero ring count | -0.85 | 0.39 |
| n5Ring | -0.13 | 5-membered ring count | -0.78 | 0.44 |
| n6ARing | -0.21 | 6-membered aromatic ring count | -0.13 | 0.90 |
| n6HRing | 0.15 | 6-membered hetero ring count | -0.87 | 0.38 |
| NaaaC | -0.44 | number of aaaC | 0.12 | 0.91 |
| NaaN | -0.28 | number of aaN | -0.08 | 0.94 |
| NaaNH | -0.79 | number of aaNH | -2.27 | 0.02 |
| NaasC | -0.25 | number of aasC | -0.45 | 0.65 |
| NaasN | 0.03 | number of aasN | -0.93 | 0.35 |
| nAcid | -0.33 | acidic group count | -1.16 | 0.25 |
| naHRing | 0.26 | aromatic hetero ring count | -1.10 | 0.27 |
| nAHRing | -0.13 | aromatic hetero ring count | -0.43 | 0.67 |
| nARing | 0.15 | aromatic ring count | 0.02 | 0.98 |
| nAromAtom | 0.46 | aromatic atoms count | -0.67 | 0.50 |
| nAtom | -0.36 | number of all atoms | -1.52 | 0.13 |
| nBase | 0.14 | basic group count | -1.73 | 0.08 |
| nBondsD | -0.25 | number of double bonds in non-kekulized structure | -1.42 | 0.16 |
| nBr | 18.28 | number of Br atoms | -0.64 | 0.52 |
| nBridgehead | 0.07 | number of bridgehead atoms | -0.33 | 0.74 |
| nC | -0.55 | number of C atoms | -1.39 | 0.16 |
| nCl | 29.36 | number of Cl atoms | 1.13 | 0.26 |
| NddsN | -0.81 | number of ddsN | 1.16 | 0.24 |
| NddssS | -0.39 | number of ddssS | -1.43 | 0.15 |
| NdsCH | 0.41 | number of dsCH | -0.03 | 0.98 |
| NdssC | 0.28 | number of dssC | -0.26 | 0.80 |
| nF | 31.04 | number of F atoms | -0.06 | 0.95 |
| nFRing | -0.22 | fused ring count | -0.81 | 0.42 |
| nHBAcc | -0.15 | number of hydrogen bond acceptor | -1.38 | 0.17 |
| nHBDon | 0.22 | number of hydrogen bond donor | -0.15 | 0.88 |
| nHetero | -1.43 | number of hetero atoms | -0.80 | 0.42 |
| nHRing | 0.13 | hetero ring count | -1.18 | 0.24 |
| nN | 0.97 | number of N atoms | -0.28 | 0.78 |
| nO | 1.39 | number of O atoms | -1.14 | 0.25 |
| nRot | -0.01 | rotatable bonds count | -0.56 | 0.58 |
| nS | -2.33 | number of S atoms | -0.77 | 0.44 |
| NsCH3 | 0.83 | number of sCH3 | -0.97 | 0.33 |
| NsNH2 | 0.54 | number of sNH2 | -0.27 | 0.78 |
| NsOH | 0.05 | number of sOH | -0.45 | 0.65 |
| NssCH2 | 0.80 | number of ssCH2 | -0.15 | 0.88 |
| NssNH | 0.26 | number of ssNH | 1.91 | 0.06 |
| NssO | -0.67 | number of ssO | -0.21 | 0.83 |
| NsssCH | 0.77 | number of sssCH | -0.63 | 0.53 |
| NsssN | 0.01 | number of sssN | -1.26 | 0.21 |
| NssssC | 0.77 | number of ssssC | 0.59 | 0.56 |
| nX | -31.55 | number of halogen atoms | 0.69 | 0.49 |
| PEOE_VSA1 | 0.05 | MOE Charge VSA Descriptor 1 (-inf < x < -0.30) | -0.74 | 0.46 |
| PEOE_VSA10 | 0.02 | MOE Charge VSA Descriptor 10 ( 0.10 <= x < 0.15) | -1.72 | 0.08 |
| PEOE_VSA11 | 0.02 | MOE Charge VSA Descriptor 11 ( 0.15 <= x < 0.20) | -0.18 | 0.86 |
| PEOE_VSA12 | 0.01 | MOE Charge VSA Descriptor 12 ( 0.20 <= x < 0.25) | 0.91 | 0.36 |
| PEOE_VSA13 | 0.00 | MOE Charge VSA Descriptor 13 ( 0.25 <= x < 0.30) | 0.60 | 0.55 |
| PEOE_VSA2 | 0.05 | MOE Charge VSA Descriptor 2 (-0.30 <= x < -0.25) | 0.85 | 0.39 |
| PEOE_VSA3 | 0.03 | MOE Charge VSA Descriptor 3 (-0.25 <= x < -0.20) | -2.23 | 0.03 |
| PEOE_VSA4 | 0.00 | MOE Charge VSA Descriptor 4 (-0.20 <= x < -0.15) | 0.14 | 0.89 |
| PEOE_VSA5 | 0.03 | MOE Charge VSA Descriptor 5 (-0.15 <= x < -0.10) | 0.16 | 0.87 |
| PEOE_VSA6 | 0.03 | MOE Charge VSA Descriptor 6 (-0.10 <= x < -0.05) | 0.76 | 0.45 |
| PEOE_VSA7 | 0.05 | MOE Charge VSA Descriptor 7 (-0.05 <= x < 0.00) | -1.48 | 0.14 |
| PEOE_VSA8 | 0.05 | MOE Charge VSA Descriptor 8 ( 0.00 <= x < 0.05) | 0.12 | 0.91 |
| PEOE_VSA9 | 0.04 | MOE Charge VSA Descriptor 9 ( 0.05 <= x < 0.10) | -0.21 | 0.83 |
| SaaO | -0.23 | sum of aaO | 0.66 | 0.51 |
| SaasC | -0.30 | sum of aasC | -0.64 | 0.52 |
| SdCH2 | 0.22 | sum of dCH2 | 0.84 | 0.40 |
| SdNH | -0.02 | sum of dNH | 0.41 | 0.68 |
| SdS | 0.02 | sum of dS | 0.63 | 0.53 |
| SdsN | 0.08 | sum of dsN | -0.42 | 0.68 |
| SdssC | -0.10 | sum of dssC | 0.95 | 0.34 |
| SdsssP | -0.05 | sum of dsssP | 1.49 | 0.14 |
| SlogP_VSA1 | 0.01 | MOE logP VSA Descriptor 1 (-inf < x < -0.40) | 1.14 | 0.26 |
| SlogP_VSA10 | -0.03 | MOE logP VSA Descriptor 10 ( 0.40 <= x < 0.50) | 1.11 | 0.27 |
| SlogP_VSA11 | 0.00 | MOE logP VSA Descriptor 11 ( 0.50 <= x < 0.60) | 0.99 | 0.32 |
| SlogP_VSA2 | 0.05 | MOE logP VSA Descriptor 2 (-0.40 <= x < -0.20) | -1.32 | 0.19 |
| SlogP_VSA3 | 0.03 | MOE logP VSA Descriptor 3 (-0.20 <= x < 0.00) | -2.60 | 0.01 |
| SlogP_VSA4 | 0.01 | MOE logP VSA Descriptor 4 ( 0.00 <= x < 0.10) | -0.21 | 0.84 |
| SlogP_VSA7 | 0.00 | MOE logP VSA Descriptor 7 ( 0.20 <= x < 0.25) | 1.02 | 0.31 |
| SMR_VSA4 | -0.05 | MOE MR VSA Descriptor 4 ( 2.24 <= x < 2.45) | 0.27 | 0.78 |
| SMR_VSA6 | -0.01 | MOE MR VSA Descriptor 6 ( 2.75 <= x < 3.05) | -0.57 | 0.57 |
| SMR_VSA9 | -0.01 | MOE MR VSA Descriptor 9 ( 3.80 <= x < 4.00) | 0.72 | 0.47 |
| SsI | -0.21 | sum of sI | -0.13 | 0.90 |
| SsSH | 0.17 | sum of sSH | 0.00 | 1.00 |
| SssS | 0.18 | sum of ssS | -0.01 | 0.99 |
| SsssCH | 0.00 | sum of sssCH | -0.34 | 0.74 |
| StCH | 0.10 | sum of tCH | 0.41 | 0.68 |
| StN | 0.06 | sum of tN | -1.08 | 0.28 |
| StsC | -0.02 | sum of tsC | -1.19 | 0.23 |
| VSA_EState1 | 0.08 | VSA EState Descriptor 1 (-inf < x < 4.78) | -1.09 | 0.28 |
| VSA_EState2 | 0.00 | VSA EState Descriptor 2 ( 4.78 <= x < 5.00) | -1.04 | 0.30 |
| VSA_EState4 | 0.03 | VSA EState Descriptor 4 ( 5.41 <= x < 5.74) | -0.27 | 0.79 |
| VSA_EState7 | 0.01 | VSA EState Descriptor 7 ( 6.07 <= x < 6.45) | -0.09 | 0.93 |
| VSA_EState8 | -0.02 | VSA EState Descriptor 8 ( 6.45 <= x < 7.00) | 0.09 | 0.93 |
| VSA_EState9 | 0.08 | Descriptor 9 ( 7.00 <= x < 11.00) | 0.46 | 0.65 |
| WPath | 0.00 | Wiener index | 0.45 | 0.65 |
| Xc-3dv | -0.29 | 3-ordered Chi cluster weighted by valence electrons | -1.02 | 0.31 |
| Xc-5d | 0.52 | 5-ordered Chi cluster weighted by sigma electrons | -0.43 | 0.67 |
| Xc-5dv | -0.17 | 5-ordered Chi cluster weighted by valence electrons | -0.23 | 0.82 |
| Xch-7d | 0.01 | 7-ordered Chi chain weighted by sigma electrons | -0.09 | 0.92 |

**Table S2. Evaluation of estimated Linear and Random Forest models for aqueous solubility predictions with outliers removed using the Molecular-Descriptors Method**

|  | **Training set** | | | **Test set** | | |
| --- | --- | --- | --- | --- | --- | --- |
|  | **R^2^** | **RMSE** | **MAE** | **R^2^** | **RMSE** | **MAE** |
| RF | 0.98 | 0.25 | 0.16 | 0.88 | 0.62 | 0.40 |
| MLR | 0.80 | 0.80 | 0.60 | 0.81 | 0.80 | 0.60 |

**Table S3. Evaluation of estimated Linear and Random Forest models for aqueous solubility predictions with outliers removed using the Morgan-Fingerprint method with key 2048 features**

|  |  | **Training set** | | | **Test set** | | |
| --- | --- | --- | --- | --- | --- | --- | --- |
|  |  | **R^2^** | **RMSE** | **MAE** | **R^2^** | **RMSE** | **MAE** |
| **2048 features** | RF | 0.96 | 0.34 | 0.22 | 0.82 | 0.80 | 0.55 |
|  | MLR | 0.85 | 0.70 | 0.53 | 0.67 | 1.09 | 0.79 |

**Table S4. List of the top 50 features in Morgan-Fingerprint with positive regression coefficients in predicting the aqueous solubility**

| **Bit** | **MLR Coefficients** |  |
| --- | --- | --- |
| 572 | 1.942373106 | 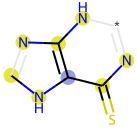 |
| 174 | 1.820302887 | 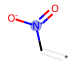 |
| 177 | 1.417542128 | 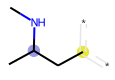 |
| 1477 | 1.315879525 | 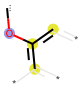 |
| 602 | 1.294722445 | 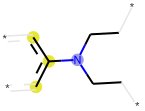 |
| 1553 | 1.186257392 | 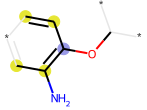 |
| 1122 | 1.163760634 | 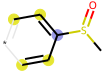 |
| 1201 | 1.161603413 | 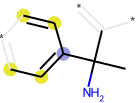 |
| 1003 | 1.14832735 | 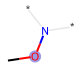 |
| 996 | 1.134916083 | 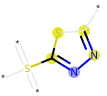 |
| 1509 | 1.126626247 | 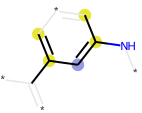 |
| 869 | 1.112410306 | 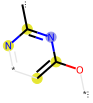 |
| 1826 | 1.080667589 | 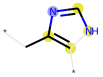 |
| 346 | 1.077496824 | 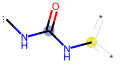 |
| 408 | 1.062644442 | 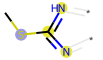 |
| 1642 | 1.058380193 | 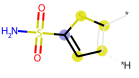 |
| 1908 | 1.053845686 | 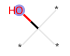 |
| 1340 | 1.04788816 | 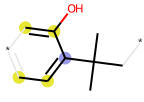 |
| 812 | 1.045203013 | 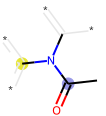 |
| 1189 | 1.041047176 | 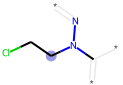 |
| 1993 | 1.035241094 | 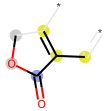 |
| 1439 | 1.032912859 | 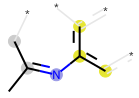 |
| 489 | 0.989373665 | 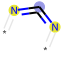 |
| 15 | 0.972541666 | 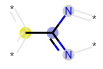 |
| 818 | 0.970794129 | 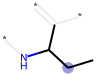 |
| 1777 | 0.95572054 | 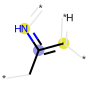 |
| 741 | 0.954676287 | 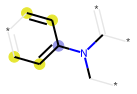 |
| 981 | 0.953941765 | 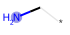 |
| 925 | 0.939359895 | 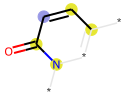 |
| 1054 | 0.935107995 | 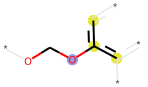 |
| 1829 | 0.918829537 | 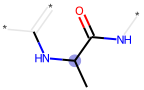 |
| 807 | 0.917207354 | 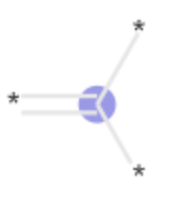 |
| 1813 | 0.915208533 | 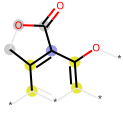 |
| 355 | 0.908530185 | 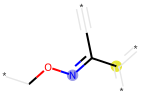 |
| 1682 | 0.886132928 | 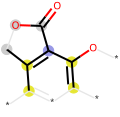 |
| 730 | 0.884783269 | 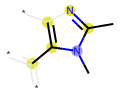 |
| 551 | 0.883015652 | 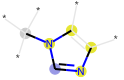 |
| 1628 | 0.881740392 | 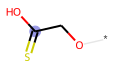 |
| 170 | 0.880438039 | 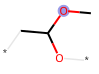 |
| 865 | 0.865629552 | 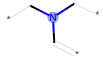 |
| 386 | 0.863458012 | 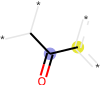 |
| 1449 | 0.857019941 | 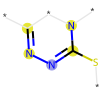 |
| 1902 | 0.853812295 | 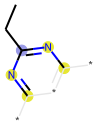 |
| 1085 | 0.842580402 | 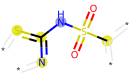 |
| 436 | 0.840357642 | 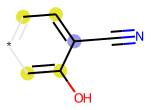 |
| 559 | 0.831421188 | 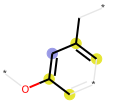 |
| 1059 | 0.826214288 | 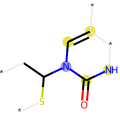 |
| 1654 | 0.824700866 | 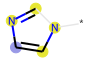 |
| 1776 | 0.824643161 | 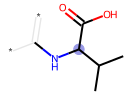 |
| 153 | 0.821925147 | 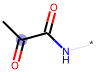 |

**Table S5. Performance of Molecular-Descriptors and Morgan-Fingerprint Methods in Predicting the Aqueous Solubility for 32 Compounds in the “Solubility Challenge”**

| Name | logS(mol/L):  Experimental | logS(mol/L): Molecular Descriptor Method | Molecular Descriptor Method -Absolute Calculation Error | logS(mol/L): Morgan-Fingerprint Method | Morgan-Fingerprint Method-Absolute Calculation Error |
| --- | --- | --- | --- | --- | --- |
| Acebutolol | -2.67 | -1.70 | 0.97 | -2.50 | 0.165 |
| Amoxicillin | -2.03 | -2.82 | 0.79 | -1.81 | 0.220 |
| Bendroflumethiazide | -3.89 | -1.88 | 2.01 | -2.49 | 1.401 |
| Benzocaine | -2.41 | -1.62 | 0.79 | -2.43 | 0.022 |
| Benzthiazide | -4.5 | -1.83 | 2.67 | -2.89 | 1.606 |
| 2-chloromandelic_acid | -0.48 | -1.53 | 1.05 | -1.09 | 0.615 |
| Clozapine | -3.24 | -1.76 | 1.48 | -3.06 | 0.182 |
| Dibucaine | -0.7 | -1.82 | 1.12 | -2.45 | 1.752 |
| Diethylstilbestrol | -2.42 | -1.80 | 0.62 | -2.06 | 0.358 |
| Diflunisal | -2.4 | -2.04 | 0.36 | -2.17 | 0.233 |
| Dipyridamole | -2.74 | -1.37 | 1.37 | -0.53 | 2.205 |
| Ephedrine | -0.5 | -1.61 | 1.11 | -1.02 | 0.518 |
| Folic_Acid | -2.87 | -2.08 | 0.79 | -2.00 | 0.874 |
| Furosemide | -4.23 | -1.73 | 2.50 | -3.70 | 0.530 |
| Hydrochlorothiazide | -2.68 | -1.85 | 0.83 | -2.76 | 0.083 |
| Imipramine | -3.6 | -1.72 | 1.88 | -2.13 | 1.473 |
| Indomethacin | -2.94 | -1.85 | 1.09 | -2.93 | 0.015 |
| Ketoprofen | -3.21 | -2.07 | 1.14 | -2.84 | 0.375 |
| Lidocaine | -1.87 | -1.59 | 0.28 | -1.82 | 0.048 |
| Marbofloxacin | -2.5 | -1.75 | 0.75 | -1.93 | 0.572 |
| Meclofenamic_acid | -3.17 | -1.94 | 1.23 | -1.74 | 1.431 |
| Naphthoic_acid | -3.77 | -1.98 | 1.79 | -3.35 | 0.417 |
| Probenecid | -1.45 | -1.61 | 0.16 | -1.02 | 0.432 |
| Pseudoephedrine | -1.4 | -1.64 | 0.24 | -2.44 | 1.037 |
| Pyrimethamine | -4.11 | -2.10 | 2.01 | -3.84 | 0.271 |
| Salicylic_acid | -1.79 | -1.37 | 0.42 | -1.42 | 0.370 |
| Sulfamerazine | -3.12 | -1.50 | 1.62 | -2.90 | 0.225 |
| Sulfamethizole | -2.78 | -1.61 | 1.17 | -2.89 | 0.109 |
| Terfenadine | -3.8 | -1.83 | 1.97 | -1.67 | 2.129 |
| Thiabendazole | -3.24 | -1.88 | 1.36 | -2.94 | 0.300 |
| Tolbutamide | -3.3 | -1.49 | 1.81 | -3.19 | 0.111 |
| Trazodone | -3.1 | -1.47 | 1.63 | -2.44 | 0.656 |
|  |  |  | Mean=1.2 |  | Mean=0.64 |
